# HABiC: an algorithm based on the exact computation of the Kantorovich-Rubinstein optimizer for binary classification in transcriptomics

**DOI:** 10.1093/bioinformatics/btaf310

**Published:** 2025-05-19

**Authors:** Chiara Cordier, Pascal Jézéquel, Mario Campone, Fabien Panloup, Agnes Basseville

**Affiliations:** LAREMA, Univ Angers, CNRS, SFR MATHSTIC, Angers F-49000, France; Institut de Cancérologie de l’Ouest, Angers F-49000, France; Institut de Cancérologie de l’Ouest, Angers F-49000, France; CRCI2NA, Nantes Université, Univ Angers, INSERM, CNRS, Nantes F-44000, France; SIRIC ILIAD, Angers F-49000, France; Institut de Cancérologie de l’Ouest, Angers F-49000, France; SIRIC ILIAD, Angers F-49000, France; LAREMA, Univ Angers, CNRS, SFR MATHSTIC, Angers F-49000, France; Institut de Cancérologie de l’Ouest, Angers F-49000, France; Institut de Cancérologie de l’Ouest, Angers F-49000, France; SIRIC ILIAD, Angers F-49000, France

## Abstract

**Motivation:**

Machine learning analyses of molecular omics datasets largely drive the development of precision medicine in oncology, but mathematical challenges still hamper their application in the clinic. In particular, omics-based learning relies on high dimensional data with high degrees of freedom and multicollinearity issues, requiring more tailored algorithms. Here, we have developed a prediction algorithm that relies on the 1-Wasserstein distance to better capture complex relationships between variables, and that is built on a decision rule based on the exact computation of the Kantorovich-Rubinstein optimizer to increase the algorithm precision. We explored dimension reduction and aggregation methods to improve its robustness. The exact method was compared with a neural network-based approximate method, as well as with standard Euclidean distance-based classifiers.

**Results:**

Experimental results on synthetic datasets with multiple scenarios of redundant/informative variables revealed that exact and approximate methods based on Wasserstein distance outperformed state-of-the-art algorithms when class information was spread across a large number of variables. When predicting clinical or biological outcomes from transcriptomics datasets, HABiC achieved consistently higher accuracy in most situations.

**Availability and implementation:**

Python code for the HABiC classifier is available at https://github.com/chiaraco/HABiC.

## 1 Introduction

Molecular omics technologies such as genomics, transcriptomics or proteomics are now commonly used in health sciences, particularly for the development of precision medicine where tumor molecular components can be used to predict treatment response or cancer subtypes for example. These technologies generate a comprehensive profiling of a selected type of cellular molecule (DNA, RNA, or protein), with the aim of capturing a complete molecular portrait of a healthy or diseased tissue. Molecular omics data are defined as high-dimensional data, characterized by a large number of measured features ($10^{3}$ to $10^{5}$ order of magnitude) and usually a small number of observations (from $10^{1}$ to $10^{3}$ patient samples per study). In practice, the adoption of precision medicine in the clinic faces the difficulty of processing high-dimensional data to capture complex data manifolds (Berisha *et al.* 2021) and still requires the improvement of mathematical approaches for this purpose.

Machine learning algorithms (MLAs) are the reference for deciphering information from large datasets, with either supervised or unsupervised learning. In both approaches, distance metrics are a key element in the construction of MLAs, where they are used to estimate model performance during the learning process. Some metrics are calculated from the distance between points that define a space X, such as the Euclidean distance defined by $X={\mathbb{R}}^{p}$. Others are defined from the space of probabilities on X, considering a dataset as a realization of an empirical measure on X. Distances and divergence measures using probability distributions include the total variation distance, the Kullback-Lleibler divergence or the Wasserstein distance [also known as the Kantorovich-Rubinstein (KR) metric].

It has been observed that the choice of distance is a fundamental point in solving high-dimensional problems and that the Euclidean distance—the most commonly used in MLA—is not always the most efficient in this context (Beyer *et al.* 1999, Aggarwal *et al.* 2001). On another note, the Wasserstein distance, computed by solving an optimal transport problem (Villani 2009), has recently gained interest in machine learning (ML) development and big data. Concretely, this metric has been shown to have superior robustness properties compared to its peers. It has been described as providing a smooth representation of the distance between distributions in low-dimensional manifolds (Arjovsky *et al.* 2017), and as taking into account the underlying geometry of distributions rather than simply measuring the difference between densities, thereby capturing structure information often lost with other dissimilarity measures (Peyré and Cuturi 2019, Bigot 2020). Optimal transport has also been used to align representations between two domains with discrepancies in data distribution (Courty *et al.* 2017).

Due to the above-mentioned properties, optimal transport and Wasserstein distance are gaining popularity in molecular omics data analyses, particularly in unsupervised approaches such as multiomics data integration (Ahmed *et al.* 2021, Cao *et al.* 2021, Demetci *et al.* 2022, Pouryahya *et al.* 2022, Zhu *et al.* 2022), trajectory inference (Schiebinger *et al.* 2019, Gan *et al.* 2022, Qu *et al.* 2025), cell-to-cell similarity measure (Cao *et al.* 2020, Huizing *et al.* 2022), generative model (Marouf *et al.* 2020, Xiao *et al.* 2021, Kircher *et al.* 2022), and spatial mapping (Nitzan *et al.* 2019, Wang *et al.* 2024). To date, supervised Wasserstein-based models have not yet been developed with molecular omics data, but the distance has been used in deep learning (DL) as a loss function for image classification (Frogner *et al.* 2015, Flamary *et al.* 2018) and in prognosis prediction models from clinical data (Zhang *et al.* 2022).

In practice, due to computational costs, Wasserstein distance approximation is often preferred over exact distance calculation with high-dimensional data. It is generally computed using DL, which is not always the most suitable when dealing with omics data. Indeed, neural networks (NNs) can suffer from the possible presence of many subspaces during optimization steps (Zheng *et al.* 2022). Also, unlike images or language, omics data do not have a clearly defined structure and could not benefit from adapted DL architectures such as convolutional NNs or recurrent NNs. Moreover, NNs require a complex and time-consuming hyperparameter optimization, established empirically when it is not predefined (which is the case with omics data), as well as the selection of an efficient gradient penalty to enforce the Lipschitz constraint required for the Wasserstein distance (Gulrajani *et al.* 2017). Finally, it is generally difficult to obtain guarantees on the convergence of a NN and therefore to distinguish numerical error from statistical error.

To overcome this, we propose to revisit the (old) Hungarian algorithm (Kuhn 1955) which, for a given discrete optimal transport problem, performs an exact computation of the optimal coupling and of a solution (Kantorovich potentials) of the so-called *Kantorovich dual problem* [see (2) for details in materials and methods]. Hungarian algorithm can be used to compute the 1-Wasserstein distance. In such a case, the distance can be expressed through a particular Lipschitz function that we named KR optimizer [see (1)]. Since this particular function can be derived from the Kantorovich potentials, we propose in this paper a KR-optimizer built from an extension of the Hungarian algorithm [see (5)]. From a practical point of view, the Hungarian algorithm operates from a cost matrix (here, the distance between all possible pairs of class 0-class 1 observations for a binary classification) and returns the optimal pairs—and some related Kantorovich potentials—so that the total cost of the assignment is as low as possible. The Wasserstein distance is then calculated by summing the transport cost of each optimal pair. The Hungarian algorithm uses combinatorial optimization in $O(n^{3})$ time to solve the assignment problem, where *n* is the number of observations.

Here, we provided a new method of Wasserstein distance-based binary classification derived from our KR optimizer to overcome the complex configuration issues inherent in DL models. We first developed single models of the Wasserstein classifier (naive method). Then, we enhanced the stability of our algorithm by regularization procedures testing either dimensionality reduction (reduction method) or bagged strategies (ensemble method). The prediction performances of these exact algorithms were compared to an approximate version computed by NN, as well as to state-of-the-art classification algorithms. To the best of our knowledge, both computations (exact or approximate) have not been tested as a classification rule in the literature.

Given that prediction performances based on transcriptomics data are largely driven by the class to predict (Shi *et al.* 2010), our classifiers were evaluated on more or less complex classification problems, using generic synthetic datasets (which allow to create controlled scenarios) as well as diverse transcriptomics datasets addressing real-world challenges such as treatment response prediction, histological grade prediction, cell type identification from single cell analysis, or distinguishing cancer tissue from normal tissue. Wasserstein distance-based methods outperformed standard algorithms for most of the tested conditions, providing a new Wasserstein-based decision rule for high dimensional data.

## 2 Materials and methods

### 2.1 Preliminary knowledge of the Wasserstein distance

Let X be a separable Banach space (in this paper, $X=R^{p}$ with p≥1). Denote by ||.|| the related norm. Let P(X) denote the space of probability measures defined on X. Then, for q≥1, we denote

$$P_{q}(X):=\{\mu \in P(X),\int_{X}||x|{|}^{q}d\mu (x)<\infty \}.$$

For $\mu ,\nu \in P_{q}(X)$, let Γ(μ,ν) denote the set of all couplings of μ and ν, where a coupling γ is a joint probability measure on X×X whose marginals are μ and ν on the first and second factors, respectively. That is, $\int_{X}\gamma (x,y)dy=\mu (x)$ and $\int_{X}\gamma (x,y)dx=\nu (y)$.

The *q*-Wasserstein distance between two distributions μ and ν is defined as

$$W_{q}(\mu ,\nu ):={\left(\underset{\gamma \in \Gamma (\mu ,\nu )}{\text{inf}}\int_{X^{2}}||x-y|{|}^{q}d\gamma (x,y)\right)}^{1/q}$$

and in particular, their 1-Wasserstein distance, also called the Earth-Mover distance, is defined by

$$W_{1}(\mu ,\nu )=\underset{\gamma \in \Gamma (\mu ,\nu )}{\text{inf}}\int_{X^{2}}||x-y||d\gamma (x,y).$$

The Wasserstein distance is the cost of the optimal transport plan. One recalls (at least from a discrete point of view) that, γ(x,y) can be interpreted as the amount of probability mass that must be transported from *x* to *y* to transform the distributions μ into the distribution ν.

The 1-Wasserstein distance, denoted $W_{1}$, can be defined in a dual way, by the KR reformulation [see e.g. Santambrogio (2015) Proposition 1.11 and Theorem 1.39]:

(1) $$W_{1}(\mu ,\nu )=\underset{\begin{array}{c} g:X\to R,\\ ||g|{|}_{L}\leq 1 \end{array}}{\text{sup}}\int_{X}g(x)(\mu -\nu )(dx)$$

 

(2) $$=\underset{(\psi ,\phi )\in \Phi }{\text{sup}}\int_{X}\psi (x)\mu (dx)+\int_{X}\phi (y)\nu (dy)$$

where $||g|{|}_{L}$ denotes the Lipschitz constant of *g*, $\Phi =\{\psi ,\phi \in C_{b}(R^{p})\;|\;\psi (x)+\phi (y)\leq ||x-y||,\forall x,y\in X\}$ and $C_{b}$ is the set of bounded continuous functions. Some functions ϕ and ψ (not unique) which attain the supremum in (2) are usually called Kantorovich potentials. As well, a function *g* which attains the supremum in (1) will be called KR optimizer in the following.

### 2.2 Development of the new Wasserstein distance-based classifiers

Let $P_{x}$ and $P_{y}$ denote two distributions on $R^{p}$ corresponding to two classes denoted by 0 and 1, respectively. Let us denote by $g^{⋆}$ a related KR-optimizer (a function that attains the supremum in (1)). Note that such a function exists under our assumptions as soon as $\int |x|P_{x}(dx)<+\infty$ and $\int |x|P_{y}(dx)<+\infty$ (owing to the Ascoli theorem, see the beginning of the proof of Supp. Proposition 2 for details). However, uniqueness does not hold since for instance, if $g^{⋆}$ is a KR-optimizer then $g^{⋆}+c$ is also a solution for any c∈R. Nevertheless, in the following, we will usually abusively talk about $g^{⋆}$ as “the” KR-optimizer. From a Wasserstein point of view, $g^{⋆}$ can be viewed as the best function for the separation between the distributions $P_{x}$ and $P_{y}$. We thus propose to use it as a way to define the following type of decision rule for binary classification

$$f^{⋆}(t)=1_{\left\{g^{⋆}(t)>\alpha \right\}}$$

where $f^{⋆}(t)$ returns the predicted class of the input *t* and α is the decision threshold to define.

In practice, one certainly deals with some series of observations coming from $P_{x}$ and $P_{y}$, respectively. Here, we assume that one has *n i.i.d.* observations of $P_{x}$ and $P_{y}$ that we denote by $\left(x_{1},\ldots ,x_{n}\right)$ and $\left(y_{1},\ldots ,y_{n}\right)$. We also denote by ${\hat{\mathbb{P}}}_{x}=\frac{1}{n}\sum_{i=1}^{n}\delta_{x_{i}}$ and ${\hat{\mathbb{P}}}_{y}=\frac{1}{n}\sum_{i=1}^{n}\delta_{y_{i}}$ the related empirical measures. With these notations, one aims at building a predictor $\hat{f}$ defined by:

$$\hat{f}(t)=1_{\left\{\hat{g}(t)>\alpha \right\}}$$

where

$$\hat{g}\in \text{Argmax}\underset{\begin{array}{c} g:X\to \mathbb{R},\\ ||g|{|}_{L}\leq 1 \end{array}}{\text{sup}}\int_{X}g(t)({\hat{\mathbb{P}}}_{x}-{\hat{\mathbb{P}}}_{y})(dt),$$

i.e.

$$\hat{g}\in \text{Argmax}\underset{\begin{array}{c} g:X\to R,\\ ||g|{|}_{L}\leq 1 \end{array}}{\text{sup}}\frac{1}{n}\sum_{i=1}^{n}(g(x_{i})-g(y_{i})).$$

In other words, $\hat{g}$ denotes a KR-optimizer related to the 1-Wasserstein distance between ${\hat{\mathbb{P}}}_{x}$ and ${\hat{\mathbb{P}}}_{y}$. The objective of the next section is to explain how to recover $\hat{g}$.

#### 2.2.1 Function recovery

**Figure 1.**
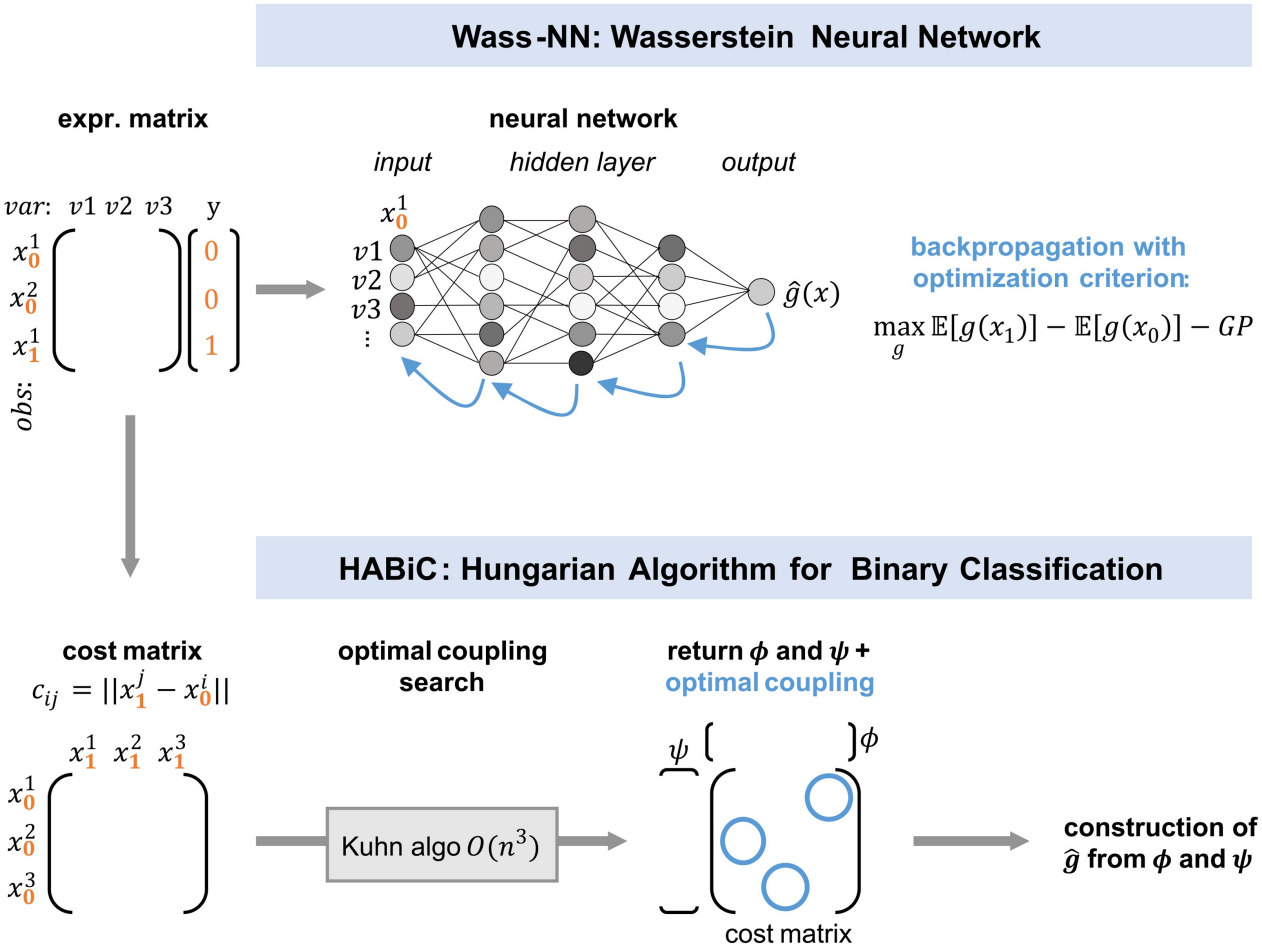
Schematic representation of the approximative and exact methods implemented to recover the *g* function related to the Wasserstein distance. GP stands for gradient penalty, var for variable, obs for observation.

Two different ways were compared to recover the $\hat{g}$ function (Fig. 1). The first one—based on the approximate method—uses the critic NN from Wasserstein GAN developed by Arjovsky *et al.* (2017) and is called the Wass-NN classifier. The second is an implementation of the exact method developed in the Kuhn version of the Hungarian algorithm (Kuhn 1955), and requires the recovery of functions ϕ and ψ found in Wasserstein distance formulation (2). This implementation is described below and is named the Hungarian algorithm for binary classification (HABiC).

In fact, this version of the Hungarian algorithm constructs throughout its execution an assignment—representing the optimally coupled observations whose precise definition is given below—as well as a solution to the dual problem (ψ and ϕ) - such that $\forall i,j\in [[1,n]],\psi (x_{i})+\phi (y_{j})\leq c_{ij}$ where *c* is the cost matrix defined by $c_{ij}=d(x_{i},y_{j})$. A pair $\left(x_{i},y_{j}\right)$ is said to be saturated when $\psi (x_{i})+\phi (y_{j})=c_{ij}$.

The operation of this algorithm is essentially based on the search for an increasing path (that is to say an alternation of saturated but unassigned pairs and assigned pairs) which makes it possible to gain an assigned pair with each inversion of the increasing path (the assigned pairs of this path become unassigned and vice versa).

To sum up, ψ and ϕ maximize $\sum_{i}\psi (x_{i})+\sum_{j}\phi (y_{j})$ under the condition that $\forall i,j\in [[1,n]],\psi (x_{i})+\phi (y_{j})\leq c_{ij}$. In that way, it solves the linear sum assignment problem, which allows the calculation of the discrete 1-Wasserstein distance. Setting $c_{ij}=||x_{i}-y_{j}||$, the $W_{1}$-distance between the empirical data distributions ${\hat{P}}_{x}$ and ${\hat{P}}_{y}$ satisfies:

(3) $$\begin{array}{c} W_{1}({\hat{P}}_{x},{\hat{P}}_{y})=\underset{\begin{array}{c} \psi ,\phi ,\\ \psi (x_{i})+\phi (y_{j})\leq c_{ij} \end{array}}{\text{sup}}\sum_{1\leq i,j\leq n}\psi (x_{i})+\phi (y_{j})\\ =\underset{\pi \in S_{n}}{\text{inf}}\sum_{1\leq i\leq n}||x_{i}-y_{\pi (i)}|| \end{array}$$

where $S_{n}$ denotes the permutations group of {1,…,n}. Denoting by $\pi^{⋆}$ an optimal permutation in (3), the pairs $\left(x_{i},y_{\pi^{⋆}(i)}\right)$ are called optimal couples related to $W_{1}({\hat{P}}_{x},{\hat{P}}_{y})$. The complementary slackness (see e.g. Chvátal 1983) asserts that for all *i*, *j*, either the $\left(x_{i},y_{j}\right)$ pair is not optimal or $\psi (x_{i})+\phi (y_{j})=c_{ij}$.

This allows us to assert that ψ and ϕ verify:

$$\psi (x_{i})=\underset{j}{\text{inf}}\;c_{ij}-\phi (y_{j})\;;\;\phi (y_{j})=\underset{i}{\text{inf}}\;c_{ij}-\psi (x_{i}).$$

It implies $\psi (x_{i})=-\underset{y_{j}}{\text{sup}}\;\{\phi (y_{j})-c_{ij}\}$ and $\phi (y_{j})=-\underset{x_{i}}{\text{sup}}\;\{\psi (x_{i})-c_{ij}\}$.

According to the link between the KR-optimizer and the pair (ψ,ϕ) from (1) and (2), a 1-Lipschitz function $\hat{g}$ will be a KR-optimizer for $W_{1}({\hat{\mathbb{P}}}_{x},{\hat{\mathbb{P}}}_{y})$ if for any *i* and *j*
 

(4) $$\hat{g}(x_{i})=\psi (x_{i})\;\text{and}\;\hat{g}(y_{j})=-\phi (y_{j}).$$

We thus define $\hat{g}$ by (4) on the dataset and need now to extend $\hat{g}$ on $R^{p}$. To this end, we introduce two natural extensions of ψ and ϕ at any *t*:

$$\tilde{\psi }(t)=-\underset{y_{j}}{\text{sup}}\;\{\phi (y_{j})-||t-y_{j}||\}\;\text{and}\;\tilde{\phi }(t)=-\underset{x_{i}}{\text{sup}}\;\{\psi (x_{i})-||x_{i}-t||\}$$

This leads to two possible $\hat{g}$ functions:

$${\hat{g}}_{1}(t)=\tilde{\psi }(t)=-\underset{y_{j}}{\text{sup}}\;\{\phi (y_{j})-||t-y_{j}||\}\;\text{and}\;{\hat{g}}_{2}(t)=-\tilde{\phi }(t)=\underset{x_{i}}{\text{sup}}\;\{\psi (x_{i})-||x_{i}-t||\}.$$

By Proposition 1, available as supplementary data at *Bioinformatics* online, for each j∈{1,2}, ${\hat{g}}_{j}$ is a KR-optimizer: ${\hat{g}}_{j}$ is 1-Lipschitz and for every *i*, *j* ${\hat{g}}_{j}(x_{i})=\psi (x_{i})$, ${\hat{g}}_{j}(y_{j})=-\phi (y_{j})$. The functions ${\hat{g}}_{1}$ and ${\hat{g}}_{2}$ are expected to differ at any point different from known points $x_{i}$ and $y_{j}$, and the computation for each $t\in R^{p}$ is based on the distances to known points $y_{j}$ and $x_{i}$, respectively. We define the $\hat{g}$ function as the mean of ${\hat{g}}_{1}$ and ${\hat{g}}_{2}$ so that the $\hat{g}$ function will allow to take into account the distances to all known points in an equitable manner, while keeping the 1-Lipschitz constraint. This means that

(5) $$\hat{g}(t)=\frac{1}{2}(\underset{x_{i}}{\text{sup}}\;\{\psi (x_{i})-||x_{i}-t||\}-\underset{y_{j}}{\text{sup}}\;\{\phi (y_{j})-||t-y_{j}||\}).$$

This averaged extension is a way to increase the robustness of the decision rule. As with the other computations, the full algorithm can be found on https://github.com/chiaraco/HABiC including the classical computation and the extension to KR-optimizers.

To end this section, let us mention a consistency property. If we denote by ${\left({\hat{g}}^{\left(n\right)}\right)}_{n\geq 1}$, the sequence of KR-optimizers defined by (5) (depending on the number *n* of observations), ${\left({\hat{g}}^{\left(n\right)}\right)}_{n\geq 1}$ converges to the set of KR-optimizers of the true distributions $P_{x}$ and $P_{y}$. More precisely, every accumulation point of ${\left({\hat{g}}^{\left(n\right)}\right)}_{n\geq 1}$ is a KR-optimizer related to $W_{1}(P_{x},P_{y})$. Such a property is stated in Proposition 2, available as supplementary data at *Bioinformatics* online.

#### 2.2.2 Norm for $W_{1}$ calculation

Several distances were tested to compare data distribution ${\hat{P}}_{x}$ and ${\hat{P}}_{y}$. For that, we used Minkowski distance with q=1 (named Manhattan distance), q=2 (Euclidean distance), q=5 and q=10. These distances are defined as follows: for $x=(x^{1},\ldots ,x^{p})$ and $y=(y^{1},\ldots ,y^{p})$,

$$d(x,y)=||x-y|{|}_{q}={\left(\sum_{\ell =1}^{p}|x^{\ell }-y^{\ell }{|}^{q}\right)}^{\frac{1}{q}}$$

#### 2.2.3 Decision rule for α

Two methods of optimal decision rule were tested to select the best α that minimizes the classification error rate. For the first method, a part of the train dataset was reserved for the optimization of a classification criterion. Three criteria were tested with either distance to the (0,1) point in the ROC curve, Matthews correlation coefficient or accuracy score (detailed in the Performance metrics paragraph below). The second method is an aggregation method where the decision threshold was calculated by averaging the scores of the highest scores of class 0 and of the lowest scores of class 1 (to reduce the impact of outliers), using the entire train dataset. Several numbers and percentages of highest and lowest scores were tried with 1, 10, 25, 50, 100, 250, 350 and 2.5%, 5%, 10%, 25% and 100%, respectively.

#### 2.2.4 Dimensionality reduction

Two-dimension reduction methods were assessed to reduce the number of variables. The first—principal component analysis (PCA)—is a non-supervised method, where the reduced features were constructed based on the combination of variables that captures the largest variance in a dataset. The second one—partial least squares discriminant analysis (PLS-DA)—is a supervised method for which important features can be filtered based on their association to the class to predict, and is recommended when the dimension reduction aims at discrimination (Barker and Rayens 2003). In both cases, we chose the number of dimensions using the elbow method from the cumulative explained variance curve of the PCA.

#### 2.2.5 Bagging (bootstrap aggregating)

Three approaches were assessed to construct a bagged HABiC classifier. In a standard approach, we built it from 100 single Wasserstein classifiers trained on a train subset sampled at random with replacement from all observations, and $\sqrt{p}$ randomly selected features (where *p* is the variable number). In the two other approaches, a variable reduction step was added after drawing $\sqrt{p}$ variables at random, by selecting 3, 5, 10 and 20 variables of importance from it or by selecting the number of important variables that allows 70% of the total importance to be retained, either by random forest (RF) or by PLS-DA. With PLS-DA, variables of importance are defined as the ones with the highest model coefficients in absolute value. For each observation, the final prediction was calculated based on the predictions obtained from the 100 single classifiers, aggregated by majority vote.

### 2.3 Datasets

#### 2.3.1 Synthetic data

To assess the performances of the algorithms on high-dimensional classification problems with various feature-related challenges, several synthetic datasets were created using the make_classification function of the scikit-learn package (Pedregosa *et al.* 2011), which generates an arbitrary classification problem, i.e. a dataset and its associated class to predict. To mimic omics data high dimension, datasets were built with 700 observations and 10 000 artificial variables. Varying levels of classification difficulties were tested with several combinations of informative variables sharing relevant information (0, 10, 100, 1000, and 5000), redundant informative variables (0, 10, 50, 400, 800, 1600, and 5000) and non-informative variables (the remaining ones). Note that increasing the number of informative variables leads to a decrease in the information per informative variable.

#### 2.3.2 Real-world transcriptomics data

Transcriptomics data were downloaded from the public repositories cBioPortal (https://www.cbioportal.org), Gene Expression Omnibus (GEO, https://www.ncbi.nlm.nih.gov/geo), Broad Institute (https://singlecell.broadinstitute.org) or Zenodo (https://zenodo.org). Dataset characteristics are detailed in Fig. 1, available as supplementary data at *Bioinformatics* online. For each classification problem, transcriptomics features (i.e. gene expression level) were harmonized by converting probes or transcripts to gene symbol name (HUGO nomenclature), aggregating the various transcripts per gene by a median, and then filtering to retain genes common to train and external cohorts. Additionally, since HABiC relies on a linear assignment requiring pairing, a re-sampling step is necessary to achieve perfectly class-balanced train datasets. This step is not penalizing, as class rebalancing is standard in ML to prevent bias toward the majority class. Consequently, random undersampling (i.e. removing samples from the majority class by randomly picking them without replacement) was systematically performed in the train cohorts.

##### 2.3.2.1 Breast cancer grade and response prediction (bulk RNA)

Three breast cancer patient cohorts were collected and filtered based on specific criteria: transcriptomics analysis was performed prior to any treatment, patients were treated with hormone therapy, and recurrence status and standard prognostic variables (tumor histological grade, lymph node status, and tumor size) were known. The METABRIC cohort (cbioportal/brca_metabric) was used as the train dataset, and the other two as external validations: Buffa (GEO/GSE22219), from the same technology as the train population (Illumina), and Hatzis (GEO/GSE25055) from Affymetrix technology. The three cohorts have 8175 genes in common. External validation cohorts were each homogenized with the train cohort using gene quantile normalization (https://github.com/cran/CONOR/blob/master/R/gq.R). Binary classification was performed on either histological grade or recurrence, the former being a more complex problem to solve. For both endpoints, gene variables were combined with the standard prognostic features for model building (excluding histological grade when it was the endpoint). For that, prognostic features were coded as 0/1, with tumor size: T0-1/T2-4, lymph node: negative/positive, histological grade: G1-2/G3, and age <50/>50.

##### 2.3.2.2 Lung cancer versus adjacent tissue prediction (bulk RNA)

Three lung cancer patient cohorts with transcriptomics data from both cancer and normal adjacent tissue were harvested, with the largest one (Zhang, GEO/GSE40791) used as train dataset, and the other two as external validations: Hou (GEO/GSE19188), from the same technology (Affymetrix), and Seo (GEO/GSE40419) from RNAseq (Illumina). They shared 15 777 common genes. No further homogenization between cohorts was performed to test our algorithm performance with dataset variability.

##### 2.3.2.3 Breast cancer cell populations (single-cell RNA)

Two breast cancer single-cell atlas were harvested. The one from Broad Institute (SCP1039) was used as a train cohort with the nine annotated major cell types used as a class to predict. Single-cell atlas from Zenodo (Xu, Zenodo/10672250) was used as an external dataset, where the 18 annotated cell-type populations were biologically combined to match the nine classes in the train cohort. For each cohort, genes with more than 2% of observations showing zero expression values were excluded, retaining 11 118 common genes between both cohorts. To decrease the high number of cells in the external validation cohort, we randomly removed cells from cell types that were more than twice the proportion of the smallest cell type. No additional homogenization between cohorts was performed.

### 2.4 Evaluation methodology

#### 2.4.1 Performance metrics

Performances were summarized by two metrics. The area under the ROC Curve (AUC) evaluates a classification model when the threshold varies over all possible values. The AUC value ranges from 0 to 1, where 0.5 represents a random classifier. Matthew’s correlation coefficient (MCC) summarizes the confusion matrix so that the metric accounts for class imbalance in the predicted data. MCC is within the range [−1,1] where +1 indicates perfect prediction, 0 means the prediction is as good as random prediction, and −1 represents perfect inverse prediction. For each model, the class-balanced METABRIC cohort was split into a train/test cohort (70/30%) preserving the class proportion, and a 5-time cross-validation (CV) was performed. Performance measures reported for AUC and MCC are the average of the 5 CV-values ± standard deviation.

#### 2.4.2 Comparison with Wasserstein-NN classifier and benchmark classifiers

The predictive performance of the Wasserstein classifier was compared with several standard MLAs, including least absolute shrinkage and selection operator (LASSO), random forests (RF), support vector machines (SVM) and a regular NN with binary cross-entropy as a loss function (regular NN). A grid search was performed for each algorithm to identify the best tuning parameters/hyperparameters. The search parameters for LASSO were: alpha: 0, 0.2, 0.5, 0.8, 1, 10, 20; max_iter: 500, 1000, 2000. The search parameters for RF were: n_estimators: 100, 200, 500; max_depth: 2, 5, 10, none; criterion: gini, entropy; min_samples_leaf: 1, 2, 5. The search parameters for SVM were: C: 0.5, 0.8, 1, 1.2, 1.5; kernel: linear, rbf, sigmoid, poly; coef0: −1, 0, 1. The search parameters for regular NN were: hidden_layer_sizes: (300, 300, 300),(1000,500); activation: relu, logistic, tanh; solver: adam, sgd, lbfgs; batch_size: auto, 64; learning_rate_init: 0.0001, 0.01; max_iter = 1000. The search parameters for the Wasserstein-NN classifier were: hidden_layer_size: (300, 300, 300),(1000,500); activation: relu, logistic, tanh; solver: adam, sgd, lbfgs; batch_size: auto, 64; learning_rate_init: 0.0001, 0.01; max_iter = 500; lambda: 2, 10.

## 3 Results

### 3.1 Development of the HABiC classifier

**Figure 2.**
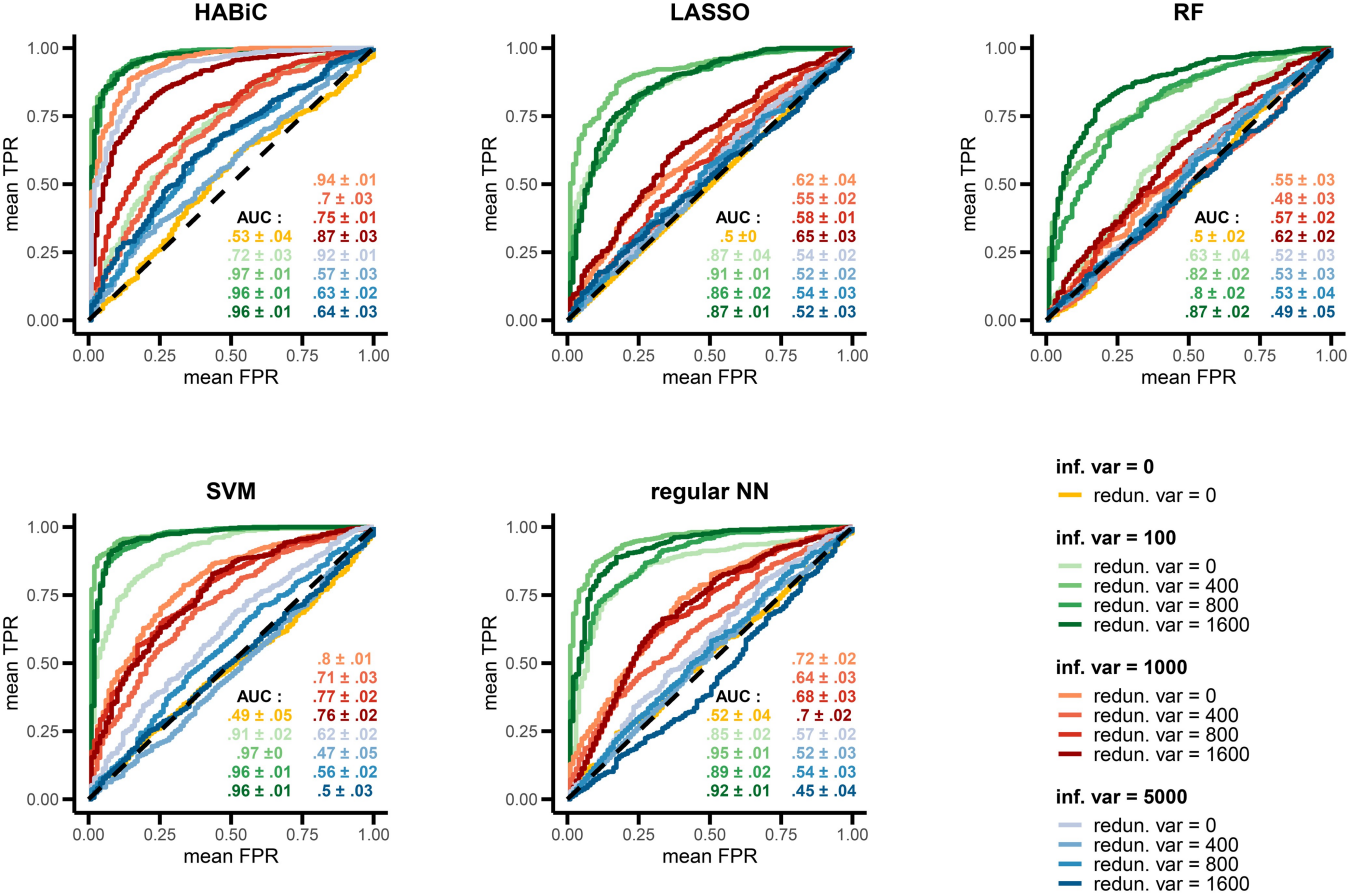
Comparison of classifier algorithm performances in synthetic datasets with various combinations of informative and/or redundant variables. ROC curves were used to compare the predictive power of the HABiC algorithm against other linear (LASSO) and non-linear (RF, SVM, regular NN) classifiers in synthetic datasets of 10 000 variables × 700 observations with various combinations of informative and redundant variables. For each tested model, after hyper-parameter tunings, ROC AUC mean values and standard deviations were calculated from a 5-fold cross-validation and indicated in the bottom right corner of each plot. Inf. var stands for informative variable, redun. var for redundant variable, TPR for true positive rate, and FPR for false positive rate.

As a first step, the Wasserstein-based classifier was implemented using the exact approach. Several norms (q=1,2,5,10) were tested to determine the most suitable probability measure for calculating the 1-Wasserstein distance with a transcriptomics dataset (Fig. 2, available as supplementary data at *Bioinformatics* online). The results indicated that 1-norm was best suited to this type of data, in accordance with other studies showing that the smaller *q* is, the less it is impacted by the high-dimensionality problem (Aggarwal *et al.* 2001).

**Figure 3.**
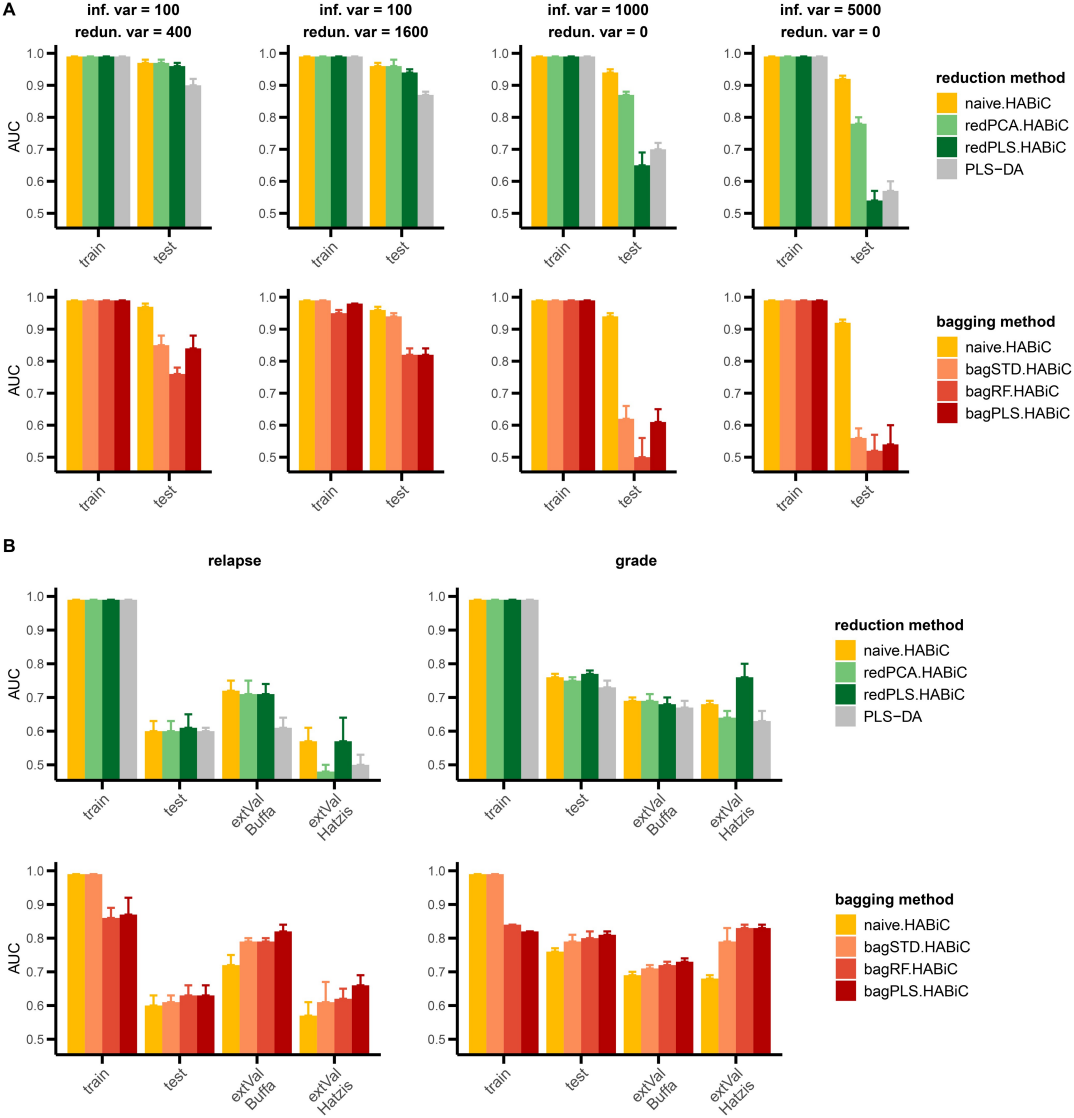
AUC prediction performance of HABiC after regularization with dimension reduction or bagging approaches in (A) synthetic datasets with various combinations of informative and/or redundant variables and (B) breast cancer transcriptomics datasets with relapse status or tumor histological grade as class to predict. Dimension reduction regularization was performed with either PCA (redPCA.HABiC) or PLS-DA (redPLS.HABiC), and PLS-DA prediction was used as a control. Bagging regularization was performed either with the standard method (bagSTD) using $\sqrt{p}$ random variable selection, with bagging using RF-based variable selection (bagRF), or with bagging using PLS-DA-based variable selection (bagPLS). Mean AUC values and standard deviations were calculated from 5-fold cross-validation. For real data, external validation (extVal) was also performed in two independent datasets (Buffa and Hatzis). Inf. var stands for informative variable, redun. var for redundant informative variable.

Synthetic datasets were then used to assess the KR-optimizer capacity to assign a prediction score. Various scenarios with different levels of collinearity and degree of freedom between variables were tested. HABiC performance was evaluated using the ROC AUC score, which enables the KR optimizer to be studied regardless of the choice of decision threshold for α. Its performances were compared with that of benchmark classifiers (LASSO-penalized logistic regressions, RF, SVM and regular NN). As observed in Fig. 2, all five algorithms performed best with the lowest proportion (1%) of explanatory variables (green curves) when combined with 400/800/1600 redundant variables, with HABiC and SVM having the best performance with an AUC of 0.96–0.97 versus an AUC of 0.80–0.95 for the others. All classifiers performed worst with negative control (0.49–0.53 AUC) or with the highest proportion (50%) of informative variables (blue curves) combined with redundant variables (0.57–0.64 for HABiC vs. 0.45–0.56 AUC for the others). Results were more disparate between classifiers in the other scenarios, but HABiC remained the best performer in the 10% informative variables scenario (red curves) with an AUC of 0.7–0.94 versus 0.48–0.8 for the others, as well as with datasets without collinearity (0.72–0.94 AUC vs. 0.52–0.91 AUC for others). Performance in a small sample size context (40, 60 or 100 observations) was also tested with HABiC and SVM algorithms using the synthetic dataset scenario for which they both achieved the best results (1% informative variables +400 redundant variables). The results indicated that both HABiC and SVM performed less well as the sample size decreased, but HABiC still performed best in low-sampling datasets (Fig. 3, available as supplementary data at *Bioinformatics* online).

**Figure 4.**
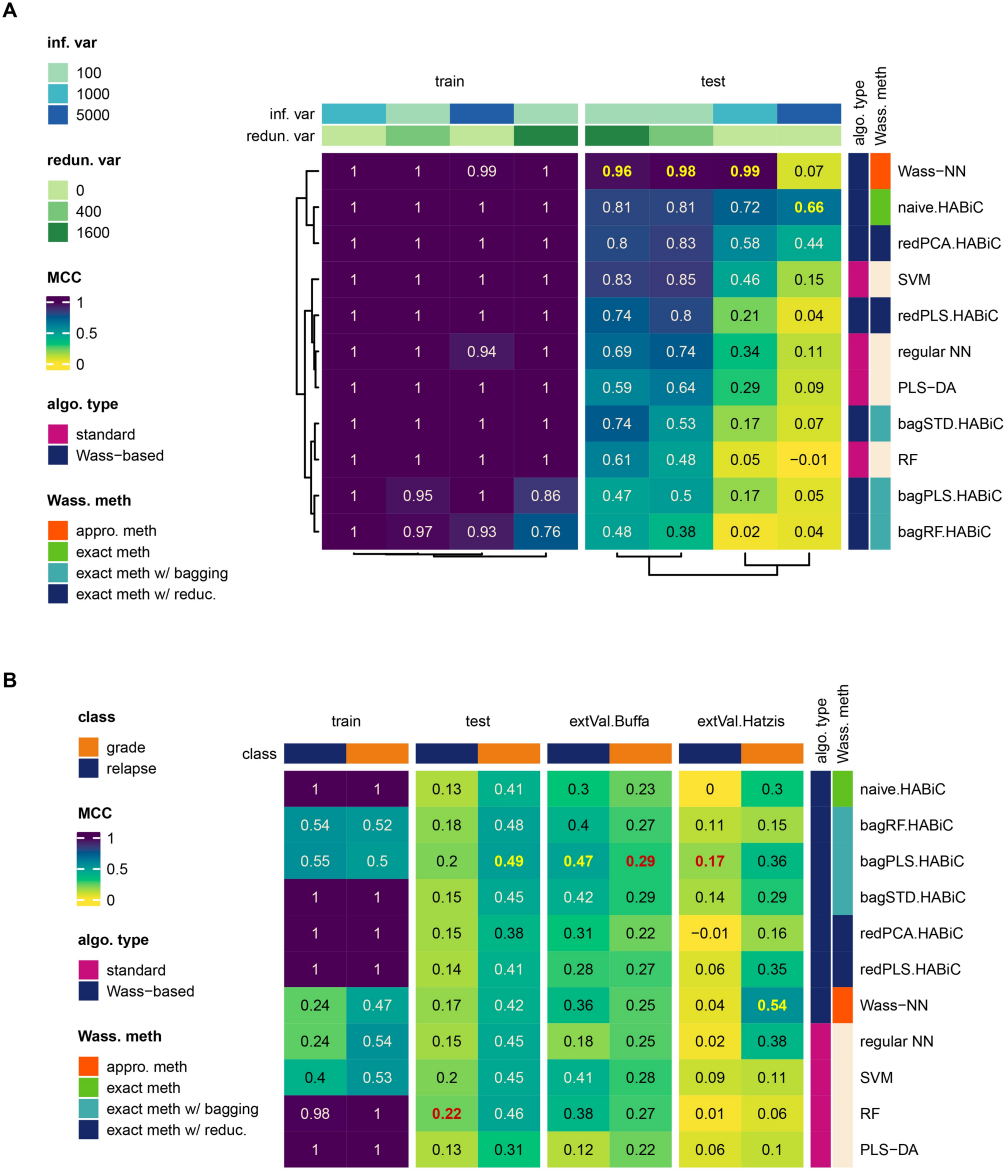
Heatmap of MCC performance comparison between classification algorithms in (A) synthetic datasets with various combinations of informative and/or redundant variables and (B) breast cancer transcriptomics datasets with relapse status or tumor histological grade as class to predict. Several standard classifiers (PLS-DA, RF, SVM, and regular NN) were compared to Wasserstein distance-based classifiers implemented with an exact approach (naive HABiC, reduction-regularized HABiC and bagging-regularized HABiC) or an approximate approach (Wass-NN). Mean MCC values were calculated using 5-fold cross-validation. Results were hierarchically clustered with synthetic datasets but not with transcriptomics datasets. For transcriptomics data, external validation (extVal) was also performed on two independent datasets (Buffa and Hatzis). For all validation results, the best model per dataset was highlighted (one per column). Inf. var stands for informative variable, redun. var for redundant informative variable, meth for method, and Wass for Wasserstein.

To assess the overfitting of the HABiC models, we compared model predictions in train versus test population and observed strong overfitting. The same overfitting was observed with SVM in most cases (Fig. 4A, available as supplementary data at *Bioinformatics* online). Nevertheless, when HABiC and SVM were trained on a negative control (i.e. synthetic datasets without informative variables for which classes were randomly assigned), when the variable dimension was increased, the HABiC models showed increasing classification performance in the train data (whereas this phenomenon was only visible for SVM from dimension 5000 onwards), but all models remained nonspecific with the test data, as desired (Fig. 4B, available as supplementary data at *Bioinformatics* online).

**Figure 5.**
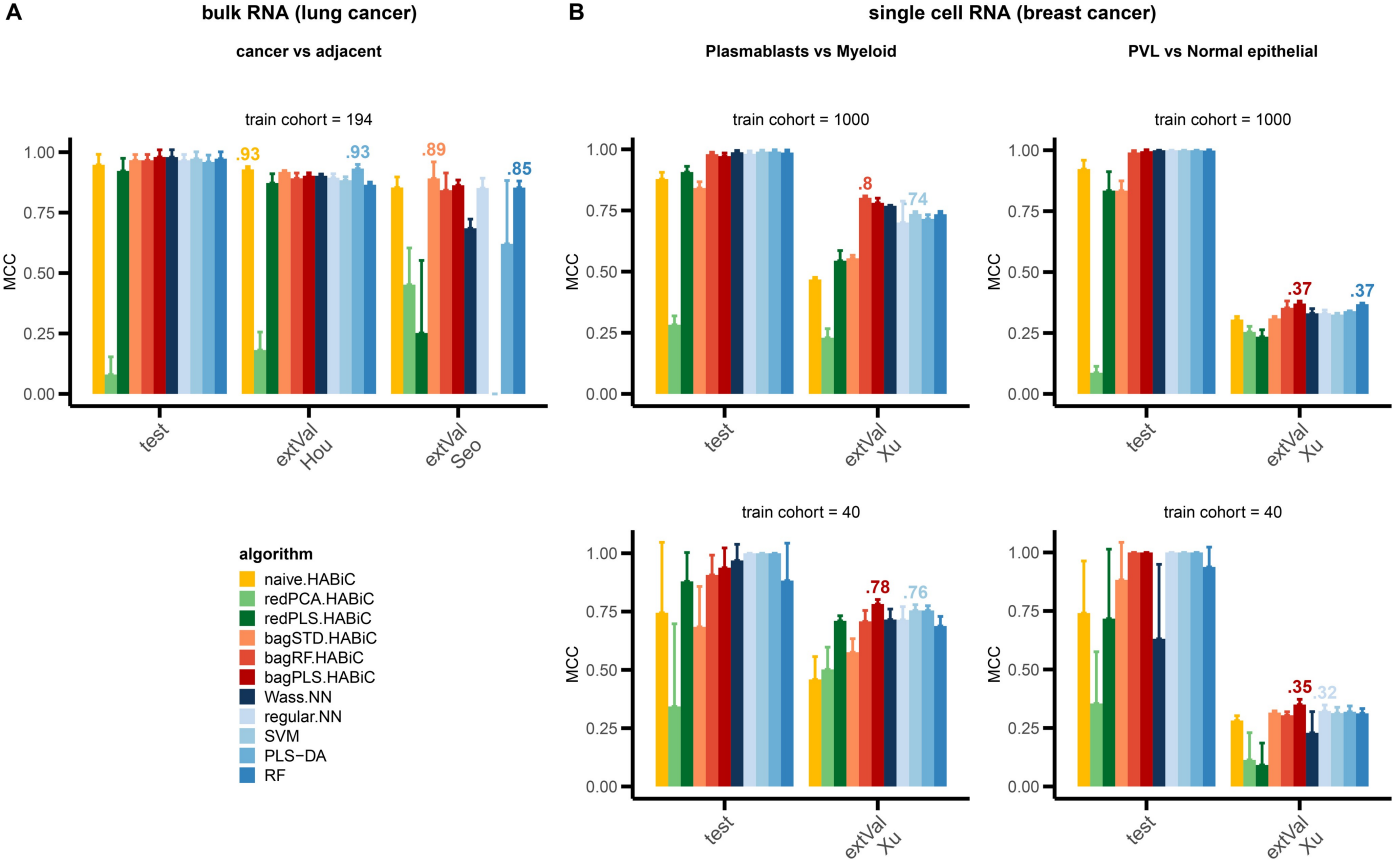
MCC performance comparison between classification algorithms in other real datasets in (A) bulk transcriptomics from lung cancer with ‘cancer’ vs. ‘adjacent tissue’ as classes to predict, and (B) single-cell transcriptomics from breast cancer, with either ‘plasmablast’ vs. ‘myeloid cells’ or ‘perivascular like (PVL)’ versus ‘normal epithelial cells’ as classes to predict. For bulk analysis, external validation (extVal) was performed in two independent datasets: the first one (Hou) from the same microarray plaform (Affymetrix U133Plus) and the second one (Seo) from RNAseq. For single-cell analysis, either 1000 or 40 cells were used as class-balanced train cohorts. Several standard classifiers (PLS-DA, RF, SVM, and regular NN) were compared to Wasserstein distance-based classifiers implemented with an exact approach (naive HABiC, reduction-regularized HABiC and bagging-regularized HABiC) or an approximate approach (Wass-NN). Mean MCC values were calculated using 5-fold cross-validation. For each external validation, the best model for each algorithm type (Wasserstein-based or standard algorithms) was annotated above the bar.

Finally, the decision threshold method was selected after testing several aggregation and optimization approaches. The results (Fig. 5, available as supplementary data at *Bioinformatics* online) indicated that averaging scores with 100% of the train set was the most advantageous method as it presents a good balance between speed of execution and reduced impact of outliers. In subsequent analyses, both AUC and MCC metrics will be used to present algorithm performances to confirm the effectiveness of the decision rule for α.

Overall, these results indicated that HABiC was globally the best achiever in synthetic datasets based on the number of scenarios in which it is most effective. Our analysis also confirmed a performance dependency inversely linked to the number of informative variables, underlining the need to test the feature regularization step to increase HABiC performance and reduce overfitting.

### 3.2 Regularization of HABiC

Two regularization approaches were tested to decrease overfitting and further improve HABiC in both synthetic and real datasets. For synthetic datasets, the four scenarios showing the best performance of naive HABiC in Fig. 2 were used. Firstly, dimension reduction was performed to reduce the number of informative variables and redundancy, using either a supervised or non-supervised method. To select the most appropriate threshold for dimension reduction, the PCA-based elbow method (based on cumulative explained variance plot) was used for both synthetic and real datasets (Fig. 6, available as supplementary data at *Bioinformatics* online). The results led us to keep 100 variables for transcriptomics data, and 100, 100, 250 and 350 variables for synthetic data with 100/400, 100/1600, 1000/0, and 5000/0 informative/redundant variables, respectively. Secondly, ensemble methods were tested, in which the predictions of several naive classifiers were grouped together to increase prediction robustness. We applied bagging methods, in which independent models were derived from bootstrap samples (see details in Section 2.2.5). Three versions were evaluated, with either a random selection of variables for each classifier, or random selection followed by subsequent filtering based on PLS-DA or RF. For methods with additional filtering, several variable selection thresholds were evaluated. As observed in Fig. 7, available as supplementary data at *Bioinformatics* online with real datasets, varying the additional filtering threshold had an impact on the performance of regularized HABiC, with an overall performance improvement for the lowest number of filtered variables (*n* = 3).

Comparing all regularization methods in the synthetic datasets (Fig. 3A for AUC and Fig. 8A, available as supplementary data at *Bioinformatics* online for MCC), we observed that all the regularized HABiCs performed worse than the naive classifier. The AUC and MCC metrics gave the same pattern of results, confirming the accuracy of the decision threshold method. In the transcriptomics datasets, we noticed that the AUC results for external validation (Fig. 3B) did not follow the same pattern as with the MCC metrics (Fig. 8B, available as supplementary data at *Bioinformatics* online), highlighting the importance of appropriate metrics when using unbalanced datasets, as here with external validation. Knowing that unbalanced datasets are correctly penalized with MCC, but that the correction is almost negligible with AUC (Chicco and Jurman 2023), we focused on MCC results for real data analysis (Fig. 8B, available as supplementary data at *Bioinformatics* online). We observed that the reduction methods showed little or no improvement over the naive method. Nevertheless, most of the bagged HABiCs showed an improved efficiency that was retained in the external validation prediction, with the best results obtained with the PLS-DA-bagged HABiCs.

Taken together, our results suggest that both naive and regularized HABiCs should be tested during model learning to obtain the best model, since naive HABiC performed best in less complex datasets (synthetic datasets), while PLS-DA-regularized HABiC was the best achiever in more complex datasets (real datasets).

### 3.3 Comparison with standard methods

Comparison of the HABiC algorithms with a selection of state-of-the-art classifiers (RF, PLS-DA, SVM and regular NN) was first performed in four scenarios of synthetic datasets and real-world transcriptomics datasets (same datasets as in Fig. 3). We also included Wasserstein-NN models to estimate the efficiency of the exact versus the approximate method (Fig. 4). For synthetic datasets, MCC results were compiled into a hierarchically clustered heatmap (Fig. 4A), highlighting (and confirming) the fact that all algorithms performed better in datasets with less informative variables (100) when combined with redundant variables (see top annotations). The naive HABiC proved the most consistent in terms of performances across informative/redundant ratio variations, and was even more efficient in the 5000/0 informative/redundant variable scenario, while Wasserstein-NN performed by far the best in the other three variable scenarios. For the real transcriptomics datasets (presented in a non-clustered heatmap in Fig. 4B), all algorithms showed roughly equal performance in the test set, with RF performing best for relapse prediction (0.22 MCC) and PLS-DA-bagged HABiC for grade prediction (0.49 MCC). Interestingly, in external validation, PLS-DA-bagged HABiC showed slightly but consistently better performances (3 out of 4 conditions tested), and Wasserstein-NN stood out strongly for the remaining condition. Furthermore, the performances obtained with synthetic datasets containing 1000 or 5000 informative variables (designed to have a reduced class-related information per variable as the number of variables increases) were in the same range as those observed in transcriptomics datasets, probably because the latter are known to also contain a large number of informative variables sharing information.

When comparing execution times between all algorithms (Table 1), the majority of HABiC methods required much less training time than other methods, with the best results for PLS-DA- and standard-bagged HABiC. In fact, with the exception of the RF bagging method, no time-consuming parameterization is required, leading to a considerable saving of time. HABiC models necessitated slightly more execution time for prediction (0.1–3.32s versus 0.006–0.51s for others).

**Table 1.** Execution times for the tested classifiers, in ascending order of fitting time. Meantime values were calculated from 5-fold cross-validation.^a^

Method	Execution time (in seconds)		
	Param. opti.	Algo. fitting	Test predict.
bagSTD.HABiC	–	0.06±0.0007	3.09±0.02
bagPLS.HABiC	–	0.7±0.02	2.35±0.02
redPCA.HABiC	–	1.15±0.12	0.11±0.01
PLS-DA	–	1.65±0.03	0.05±0.0006
bagRF.HABiC	321±1.81	1.77±0.62	2.35±0.02
RF	634	1.98±0.03	0.14±0.004
redPLS.HABiC	–	2.06±0.11	0.1±0.003
naive.HABiC	–	2.49±0.42	3.32±0.24
SVM	217	6.34±0.15	0.51±0.008
Wass-NN	18 103	42.2±1.13	0.006±0.004
regular NN	11 420	48.9±6.89	0.06±0.004

a Param. opti. stands for parameters optimization, Algo. fitting for algorithm fitting and Test predict. for test prediction.

To further assess the capacity of our algorithms and the standard ones in other real-world applications, we tested them on less complex problems, such as the classification of cancer tissue versus normal adjacent tissue in the lung (Fig. 5A), or the classification of two-cell types in single-cell analysis from breast cancer (Fig. 5B). Since 36 pairs of comparison were possible with single-cell analysis, a screening was first performed by RF on a 1000-cell train population (Fig. 9, available as supplementary data at *Bioinformatics* online) to select two pairs with different classification problem complexity. Accordingly, we selected a pair with a moderate complexity (plasmablasts versus myeloid cells, 0.71 MCC in external validation), and a second pair with the highest complexity (PVL versus normal epithelial cells, 0.35 MCC). Moreover, we also tested algorithm performance in two sample size contexts (1000 or 40 observations in a train cohort). In all applications, most models (except the reduction-regularized HABiC methods) performed very well in test cohorts (above 0.90 MCC), confirming a problem much easier to solve than the histological grade or treatment response tested in Fig. 4 (that showed a maximum of 0.49 and 0.22 MCC in test cohort, respectively). Results were more variable in external validation cohorts. In lung classification problems, results were equivalently good in the independent cohort from the same microarray platform (with equal best performance—0.93 MCC—in Wasserstein-based algorithms versus the standard ones). Performances were more disparate in the RNAseq-independent cohort, with standard-bagged HABiC showing the best results (0.89 MCC). In single-cell classifications, RF- and PLS-DA-bagged-HABiCs were the best performers on 3 out of 4 cases (with 2- to 6-point gain in MCC compared to state-of-the-art algorithms).

Overall, these results highlighted the dominant position of Wasserstein distance-based classifiers over those using Euclidean distance in high dimensional datasets, whether for real or synthetic data. The choice of an exact or approximate Wasserstein-based method is then strongly linked to variable characteristics (levels of collinearity and degree of freedom). Here, exact methods (and more particularly the bagged-HABiC) were generally more suited to highly complex transcriptomics datasets (which typically have a lot of redundancy and informative variables), while the approximate method seemed better suited to less complex datasets (synthetic data with the lowest number of informative variables).

## 4 Discussion

Predicting a patient’s response to treatment based on tumor molecular information is one of the main aims of precision medicine in oncology. Nevertheless, these prediction models are for the most part far from clinical application, and their development remains a challenging field with incremental progress. In this study, we focused on the distance used to compare two probability distributions in a transcriptomics-based classification problem and developed two Wasserstein distance-based classifiers with improved performance compared to commonly used Euclidean distance-based algorithms, particularly in datasets with the highest degree of complexity (related to collinearity and degree of freedom).

One of the steps in improving a prediction model performance is the empirical selection of the most suitable algorithm for predicting a phenotype, where a series of algorithms are tested to find the one best suited to a dataset and its own characteristics (dimensionality and complexity). Several studies have already compared the use of linear vs. nonlinear algorithms (Heil *et al.* 2023), or DL vs. standard ML algorithms (Smith *et al.* 2020) to predict a phenotype from gene expression. The results showed that nonlinear ML and DL did not always perform best with gene expression data. Even graph NNs with integration of prior biological knowledge showed inferior or comparable performance to standard ML methods (Brouard *et al.* 2024), suggesting that leverages other than linearity, learning layers or biological input need to be tested in model design for omics data. Our study shows that distance metrics are one of them.

The difficulty of using transcriptomics data for classification problems is not mainly due to the high dimensionality of the data, since even with transcriptomics datasets, algorithm performance depends first and foremost on the phenotype to be predicted. As an example, the MAQC-II study shows that many algorithms can achieve excellent performance when an easy phenotype such as patient sex is predicted (Shi *et al.* 2010). This indicates that the primary cause of performance variation is the link between the variables and the class to predict, i.e. the collinearity of the variables and/or their degree of freedom. Here, we demonstrated that all the algorithms tested—with different linearities, layer levels and distances—behaved in the same way when faced with variation in the number of informative variables, regardless of collinearity (Fig. 2). The more information is spread over a smaller number of variables, the more efficient the algorithms. Nevertheless, HABiC and Wass-NN outperformed the other MLAs as data complexity increased, underlining the fact that Wasserstein distance is best suited when class information is shared across many variables. The advantages of using Wasserstein over other distances have been seen previously with transcriptomics data for cell–cell similarity detection (Huizing *et al.* 2022) or with images for classification models (Snow and Van Lent 2018).

We also observed that the exact method (HABiC) performed better in real datasets while the approximate method (Wass-NN) performed better with synthetic datasets. In theory, exact algorithms are more accurate since they provide a mathematically exact solution contrary to approximate versions that provide a solution close to the optimal one, although not exact. Since exact algorithms are computationally expensive and time-consuming, they are usually not usable in large-scale problems and the approximate version, built to be more efficient, is often the only one usable. Nevertheless, in our case, the execution speed for the linear assignment problem depends on the number of observations but not on the number of variables, which made its application possible in transcriptomics where datasets rarely exceed 1000 patients. Moreover, hyper-parameter tuning is not required for HABiC, which further reduces its execution time compared to the NN-based solution.

There are limitations when using HABiC with transcriptomics. First, since linear assignment is based on pairing, classes must be perfectly balanced, and a rebalancing step is necessary if this is not the case. Second, there is no direct possibility for biological interpretability of HABiC-derived models, as the selection of the KR-optimizer directly relies on a minimization along the whole class of Lipschitz functions, implying that there is no direct way to extract importance variables. Nevertheless, it is possible to overcome this problem by using the leave one feature out (LOFO) importance method, where each feature is iteratively removed from the dataset, then the model is retrained without it, and model performance with/without is compared [see detailed explanation in chapter 24 of Molnar (2025)]. In addition, the Wass-NN algorithm can handle unbalanced classes, and methods for interpreting variables in NN exist, such as layer-wise relevance propagation (Montavon *et al.* 2019).

In conclusion, HABiC and Wass-NN are complementary algorithms that are suitable for classification problems with high-dimension data. This study also highlights that exact algorithms and Wasserstein distance are adapted for gene expression data analysis. In the challenging field of molecular-based precision medicine, these new pieces to the puzzle bring us closer to clinically applicable solutions.

## Supplementary Material

btaf310_Supplementary_Data

## Data Availability

Public datasets were downloaded from cBioPortal (www.cbioportal.org) or GEO (https://www.ncbi.nlm.nih.gov/geo), and all accession IDs are reported in section 2.3. Code for data preprocessing is described at https://github.com/chiaraco/HABiC and pre-processed data are accessible at https://zenodo.org/records/15091117.
