# Supplementary material for: HABiC: an algorithm based on the exact computation of the Kantorovich-Rubinstein optimizer for binary classification in transcriptomics
Source: Bioinformatics. 2025 May 19;41(6):btaf310. doi: 10.1093/bioinformatics/btaf310 (PMC12198494; doi:10.1093/bioinformatics/btaf310)
Supplement: btaf310_Supplementary_Data [file btaf310_supplementary_data.pdf]

Proofs and propositions

Proposition 1. *Let* $\hat{g}_1(t) = -\underset{y_j}{sup}\ \{\phi(y_j) - \|t - y_j\|\}$ *and* $\hat{g}_2(t) = \underset{x_i}{sup}\ \{\psi(x_i) - \|x_i - t\|\}$ *where* x_i, y_j *are training observations. The functions* $\hat{g}_k$*,* $k = 1, 2$*, are* 1*-Lipschitz continuous and satisfy*

$$\hat{g}_k(x_i) = \psi(x_i) \quad \text{and} \quad \hat{g}_k(y_j) = -\phi(y_j). \tag{1}$$

Proof Since the arguments are similar for $\hat{g}_1$ and $\hat{g}_2$, we only detail the proof for $\hat{g}_1$.
For $i_0 \in \{1, \ldots, n\}$, let $j_0 \in \{1, \ldots, n\}$ be such that (x_{i_0}, y_{j_0}) is an optimal pair, *i.e.* $\psi(x_{i_0}) + \phi(y_{j_0}) = \|x_{i_0} - y_{j_0}\|$ and $\psi(x_{i_0}) + \phi(y_j) \leq \|x_{i_0} - y_j\|$ for any $j \in \{1, \ldots, n\}$. This means that

$$\begin{aligned} \psi(x_{i_0}) &= \inf\{\|x_{i_0} - y_j\| - \phi(y_j), j\} \\ &= -\sup\{\phi(y_j) - \|x_{i_0} - y_j\|\} = \hat{g}_1(x_{i_0}). \end{aligned}$$

This corresponds to the first condition of (1). For the second one, let us remark that if there exists y_j such that $\phi(y_j) - \|y_{j_0} - y_j\| > \phi(y_{j_0}) - \|y_{j_0} - y_{j_0}\| = \phi(y_{j_0})$ then since $\psi(x_{i_0}) + \phi(y_{j_0}) = \|x_{i_0} - y_{j_0}\|$, we deduce that

$$\phi(y_j) + \psi(x_{i_0}) > \|y_{j_0} - y_j\| + \|x_{i_0} - y_{j_0}\|.$$

By the triangular inequality, this involves that

$$\phi(y_j) + \psi(x_{i_0}) > \|x_{i_0} - y_j\|,$$

which is a contradiction. Hence $\hat{g}_1(y_{j_0}) = -\phi(y_{j_0})$.

Finally, let us prove that $\hat{g}_1$ is 1-Lipschitz: for $t, t' \in \mathcal{X}$,

$$\begin{aligned} -\hat{g}_1(t) &= \sup_{y_j}\ \{\phi(y_j) - \|t - y_j\|\} \\ &\leq \sup_{y_j}\ \{\phi(y_j) + \|t - t'\| - \|t' - y_j\|\} \\ &\leq -\hat{g}_1(t') + \|t - t'\| \\ \hat{g}_1(t') &\leq \hat{g}_1(t) + \|t - t'\| \end{aligned}$$

By symmetry, $\hat{g}_1(t) \leq \hat{g}_1(t') + \|t - t'\|$. Hence $\|\hat{g}_1(t) - \hat{g}_1(t')\| \leq \|t - t'\|$.
□

In the proposition below, we denote by $\hat{g}^{(n)}$ a KR-optimizer related to the W_1-distance between the empirical distributions $\hat{P}_X = \frac{1}{n}\sum_{i=1}^{n} \delta_{X_i}$ and $\hat{P}_Y = \frac{1}{n}\sum_{i=1}^{n} \delta_{Y_i}$ where $(X_k)_{k\geq 1}$ and $(Y_k)_{k\geq 1}$ denote *i.i.d.* sequences of *r.v.* with distributions $\mathbb{P}_X$ and $\mathbb{P}_Y$ respectively. We prove that $(\hat{g}^{(n)})_{n\geq 1}$ has the expected convergence properties towards the set of KR-optimizers of the true distributions $\mathbb{P}_X$ and $\mathbb{P}_Y$.

Proposition 2. *Let* $\mathbb{P}_X$ *and* $\mathbb{P}_Y$ *denotes two probability distributions on* $\mathbb{R}^p$ *which are such that* $\mathbb{E}[|X|] < +\infty$ *and* $\mathbb{E}[|Y|] < +\infty$*. Then,*

$$\left| \frac{1}{n}\sum_{i=1}^{n} \hat{g}^{(n)}(X_i) - \frac{1}{n}\sum_{i=1}^{n} \hat{g}^{(n)}(Y_i) \right| \xrightarrow{n\to+\infty} W_1(\mathbb{P}_X, \mathbb{P}_Y)$$

almost surely. Furthermore, for any subsequence of $\hat{g}^n$*, one can extract a convergent subsequence to* g_∞*, for the topology of uniform convergence on compact sets, where* g_∞ *is a KR-optimizer of the true distributions, i.e.,*

$$|\int g_\infty d\mathbb{P}_X - \int g_\infty d\mathbb{P}_Y| = W_1(\mathbb{P}_X, \mathbb{P}_Y).$$

Proof Let $\mathrm{Lip}_1(\mathbb{R}^p)$ denote the set of 1-Lipschitz continuous functions from $\mathbb{R}^p$ to $\mathbb{R}$. We first prove the existence of a KR-optimizer for two given probabilities μ and ν. Let $(g_k)_{k\geq 1}$ be a sequence of 1-Lipschitz functions which is such that

$$\mu(g_k) - \nu(g_k) \xrightarrow{k\to+\infty} W_1(\mu,\nu).$$

At the price of replacing g_k by $g_k - g_k(0)$, we can assume that $g_k(0) = 0$ for every $k \geq 1$. Then, since $(g_k)_{k\geq 1}$ is equicontinuous as a sequence of 1-Lipschitz functions, we can deduce from Ascoli's theorem that one can extract a subsequence which converges to g for the topology of uniform convergence on compacts sets of $\mathbb{R}^p$. The function g is certainly 1-Lipschitz and satisfies $\mu(g)-\nu(g) = W_1(\mu,\nu)\}$. This is thus a KR-optimizer.

As a consequence, the sequence $(\hat{g}^n)_{n\geq 1}$ is well-defined (note that the existence of $\hat{g}^n$ was in fact provided by the Hungarian algorithm) and satisfies: for all $n \geq 1$,

$$\hat{g}^n \in \mathrm{Argmax}\left\{\frac{1}{n}\sum_{i=1}^{n} g(X_i) - g(Y_i), g \in \mathrm{Lip}_1(\mathbb{R}^p)\right\}.$$

With the same Ascoli argument, from any subsequence of $(\hat{g}^n)_{n\geq 1}$, one can extract a convergent subsequence $(\hat{g}^{n_k})_{n_k\geq 1}$ which converges to $g_\infty \in \mathrm{Lip}_1(\mathbb{R}^p)$. Finally, since by the law of large numbers and Villani, 2009 Theorem 6.8, $W_1(\frac{1}{n}\sum_{i=1}^n \delta_{X_i}, \mathbb{P}_X) \xrightarrow{n\to+\infty} 0$ and $W_1(\frac{1}{n}\sum_{i=1}^n \delta_{Y_i}, \mathbb{P}_Y) \xrightarrow{n\to+\infty} 0$, the result follows.
□

Supplementary figures

A. bulk / breast cancer

batch + reference ID	dataset	platform	relapse	median age [Q1-Q3]	gender	time period	follow-up median/end	tumor size	lymph node	histological grade	hormono-therapy
**Metabric (1903)**<br>brca_metabric	train	Illumina<br>HT-12 v3<br>(microarray)	no (1132)<br>yes (771)	62<br>[51-71]		1977-<br>2005	8.4	T0-1 (591)<br>T2-4 (1292)<br>NA (20)	Neg (992)<br>Pos (911)	G1-2 (905)<br>G3 (926)<br>NA (72)	no (729)<br>yes (1174)
**Buffa (133)**<br>GSE22219	external<br>validation	Illumina<br>humanRef-8<br>(microarray)	no (84)<br>yes (49)	57<br>[49-64]		1989-<br>1992	10 yrs<br>/10	T0-1 (41)<br>T2-4 (92)	Neg (77)<br>Pos (56)	G1-2 (94)<br>G3 (26)<br>NA (13)	yes (133)
**Hatzis (310)**<br>GSE25055	external<br>validation	Affymetrix<br>HGU133 A<br>(microarray)	no (244)<br>yes (66)	49<br>[43-58]		2000-<br>2006	2.4 yrs<br>/3	T0 (2), T1(20)<br>T2 (165), T3(74)<br>T4 (49)	Neg (87)<br>Pos (223)	G1-2 (136)<br>G3 (151)<br>NA (23)	no (131)<br>yes (178)

B. bulk / lung cancer

batch + reference ID	dataset	platform	tissue	median age [Q1-Q3]	gender	time period	clinical stage
**Zhang (194)**<br>GSE40791	train	Affymetrix<br>HGU133 plus2<br>(microarray)	adjacent (100)<br>cancer (94)	70<br>[62-75]	F (83)<br>M (111)	1997-<br>2007	1 (69) / 2 (12)<br>3 (23)
**Hou (156)**<br>GSE19188	external<br>validation	Affymetrix<br>HGU133 plus2<br>(microarray)	adjacent (65)<br>cancer (91)	NA	F (34)<br>M (100)<br>NA (22)	1992-<br>2004	NA
**Seo (164)**<br>GSE40419	external<br>validation	Illumina<br>HiSeq 2000<br>(RNAseq)	adjacent (77)<br>cancer (87)	65<br>[58-70]	F (67)<br>M (97)	2010-<br>2011	1 (55) / 2 (13)<br>3 (13) / 4 (4)<br>NA (79)

C. single cell RNAseq / breast cancer

batch + reference ID	dataset	platform	original cell number	after resampling	major cell types (already annotated)
**Broad**<br>SCP1039	train	Illumina<br>NextSeq 500	100 064	28 422	B-cells (3158), CAFs (3158), Cancer Epithelial (3158), Endothelial (3158), Myeloid (3158), Normal Epithelial (3158), Plasmablasts (3158), PVL (3158), T-cells (3158)
**Xu**<br>10672250	external<br>validation	Illumina<br>(mixed)	236 363	26 048	B-cells (3162), CAFs (3162), Cancer Epithelial (3162), Endothelial (3162), Myeloid (3162), Normal Epithelial (2333), Plasmablasts (3162), PVL (1581),T-cells (3162)

Supp. Figure 1: **Dataset presentation**. Datasets were obtained from several repositories (GEO, cBioportal, Broad Institute and Zenodo), with "Reference ID" referring to identifiers and their associated link. **A.** Three breast cancer patient cohorts were used for relapse or histological grade prediction. All types of recurrence (local, regional, distant) were included in relapse. "Q1" and "Q3" refer to lower quartile and upper quartile, respectively. Follow-ups are in years (yrs).The four categories for tumor size are defined as: T0 (no evidence of tumor) $<$ T1 $\leq$ 2 cm $<$ T2 $\leq$ 5 cm $<$ T3, and T4 has grown into the neighboring tissue. "Pos" and "Neg" stand for positive and negative, and "NA" for not available. G1-2 refers to low grade and intermediate histological grade, and G3 to high histological grade. **B.** Three lung cancer patient cohorts were used for cancer versus adjacent tissue prediction. "F" and "M" stand for female and male, respectively. **C.** Two previously annotated single cell datasets from breast cancer patients were used to develop cell type predictionmodels. CAF stands for cancer-associated fibroblast, and PVL for perivascular-like.

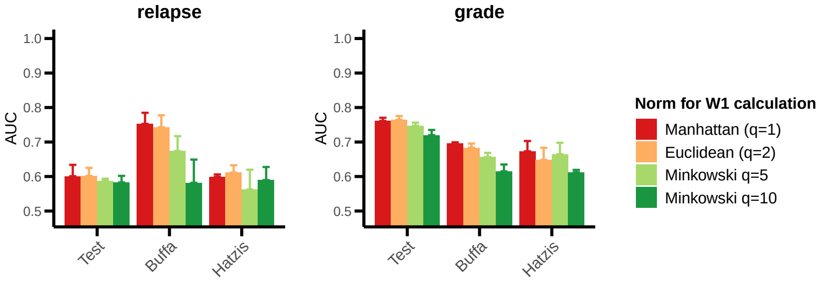

Supp. Figure 2: **Impact of norm choice on HABiC prediction performance.** The Wasserstein distance-based classifier was implemented using an exact approach by testing 4 norms ($q = 1, 2, 5, 10$) to calculate the 1-Wasserstein distance. The performance of the prediction models was then tested on real data using transcriptomic dataset (METABRIC) for the prediction of relapse or tumor histological grade. Mean AUC values and standard deviations were calculated from 3-fold cross-validation. External validation was also performed in two independent datasets (Buffa and Hatzis).

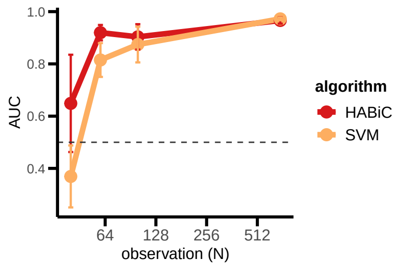

Supp. Figure 3: **Algorithm performance comparison according to the number of observation in the train set**. Synthetic datasets with 10,000 variables x various number of observations were used, with 1% informative variables + 400 redundant variables. Mean AUC values and standard deviations were calculated from 5-fold cross-validation.

Supp. Figure 4: **(next page). Overfitting assessment in HABiC versus SVM models.** ROC curves were calculated based on the train set used in Figure 2 (in which the test set is presented). In the case of overlapping lines, mean AUC values and standard deviations calculated from 5-fold cross-validation are indicated in the bottom right corner of each graph. (B.) The algorithms were trained using several synthetic negative control datasets (non-informative variables with a randomly assigned class) with 700 observations and various number of variables. The scores per observation assigned by the HABiC and SVM models are shown on the y-axis for the train and test sets, and the observations are indexed on the x-axis and colored according to initial randomly assigned class.

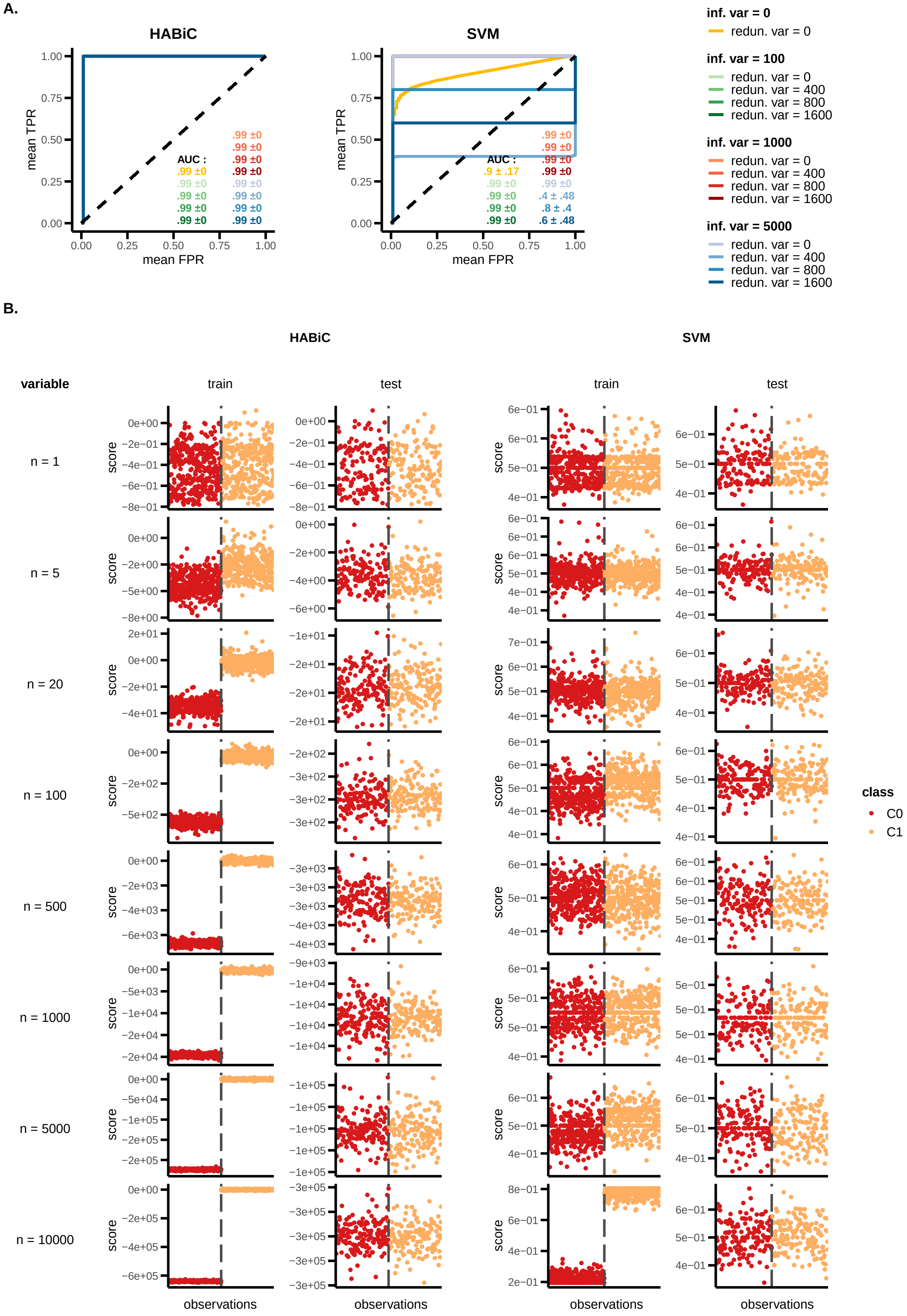
A.
HABiC
SVM
mean TPR
mean FPR
AUC :
inf. var = 0
redun. var = 0
inf. var = 100
redun. var = 0
redun. var = 400
redun. var = 800
redun. var = 1600
inf. var = 1000
redun. var = 0
redun. var = 400
redun. var = 800
redun. var = 1600
inf. var = 5000
redun. var = 0
redun. var = 400
redun. var = 800
redun. var = 1600
B.
HABiC
SVM
variable
train
test
n = 1
n = 5
n = 20
n = 100
n = 500
n = 1000
n = 5000
n = 10000
score
observations
class
C0
C1

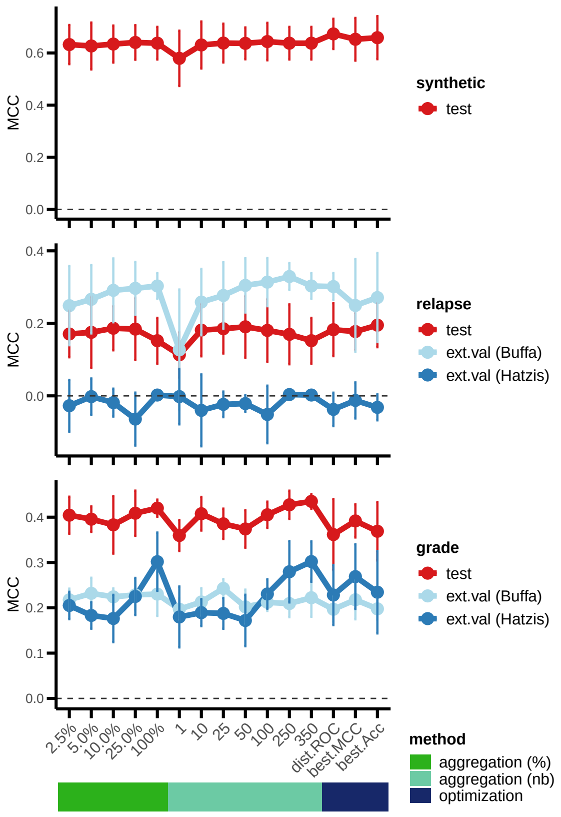

Supp. Figure 5: **Comparison of the decision threshold method for HABiC in synthetic and real datasets** with performance calculated using MCC metrics. For the synthetic data (top panel), datasets with 10,000 variables x 700 observations were used, with 1% informative variables + 400 redundant variables. For the real dataset, relapse and histological grade were predicted based on the transcriptomics dataset, and external validation (ext.val) was also performed in two independent datasets (Buffa and Hatzis). Mean MCC values (+/- standard deviations) were calculated from 5-fold cross-validation.

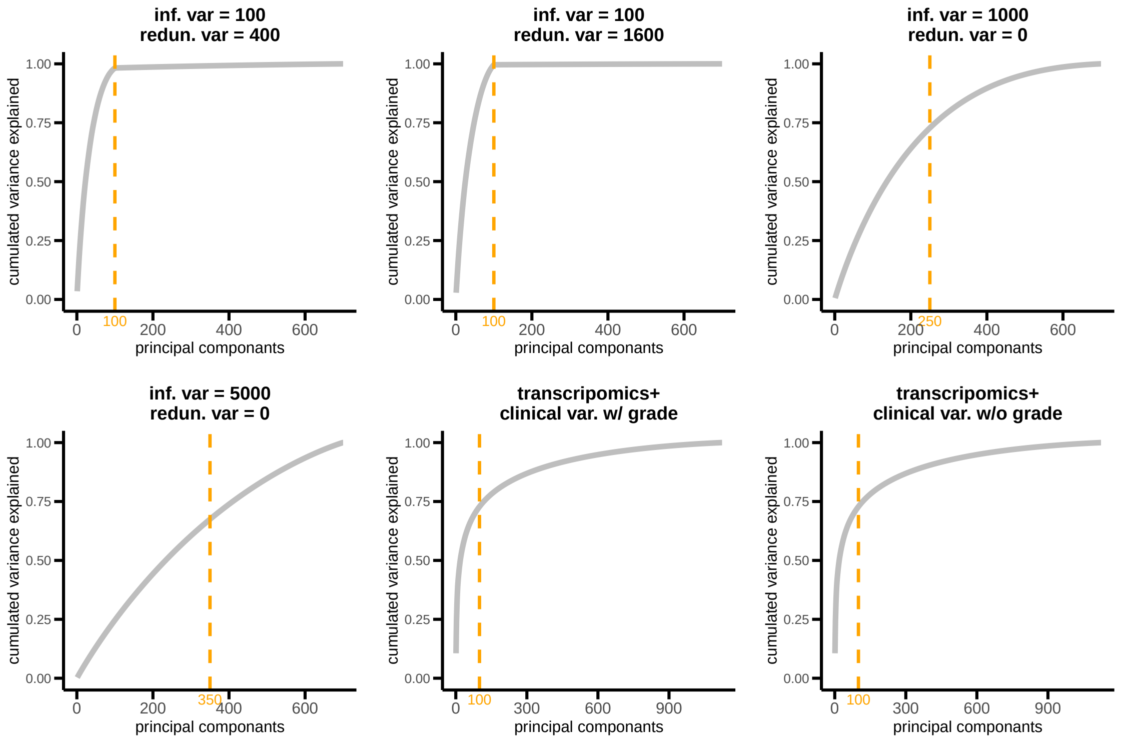

Supp. Figure 6: **Choice of the dimension reduction threshold by the elbow method based on the PCA cumulative explained variance in synthetic and real datasets**. The number of variable used for the dimension reduction was chosen according to the cumulative explained variance resulting from a PCA analysis. For the synthetic data, datasets with 10,000 variables x 700 observations were used, with four scenarios of number of informative and redundant variables. For the real datasets, PCA was performed on transcriptomic data combined with standard clinical prognostic variables including or not the histological grade. When the elbow was not clearly visible, a threshold resulting on a cumulative explained variance of at least 0.7 was used. The selected thresholds are indicated in orange on the graphs.

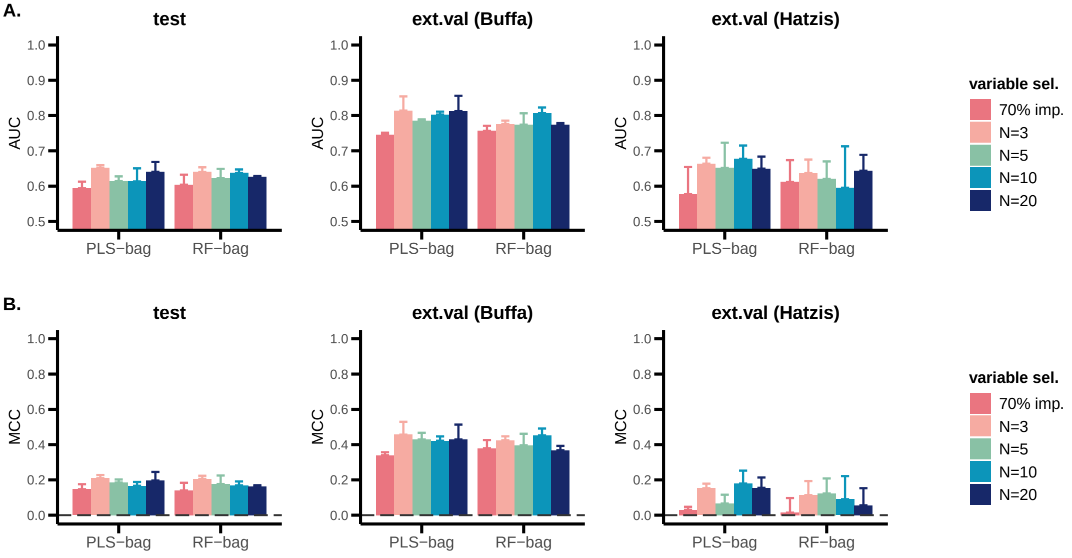

Supp. Figure 7: **PLS-DA- and RF-based variable reduction step assessment for bagging method development**. Additional variable selection steps were added in the bagging after drawing $\sqrt{p}$ variables randomly, by selecting either 3, 5, 10, 20 variables of importance from it, or the number of important variables that allows to retain 70% of the total variable importance. Relapse prediction performance based on transcriptomic dataset are presented using either AUC (A.) or MCC (B.) metrics. Mean values +/- standard deviations were calculated from 5-fold cross-validation, and external validation (ext.val) was also performed in two independent datasets (Buffa and Hatzis).

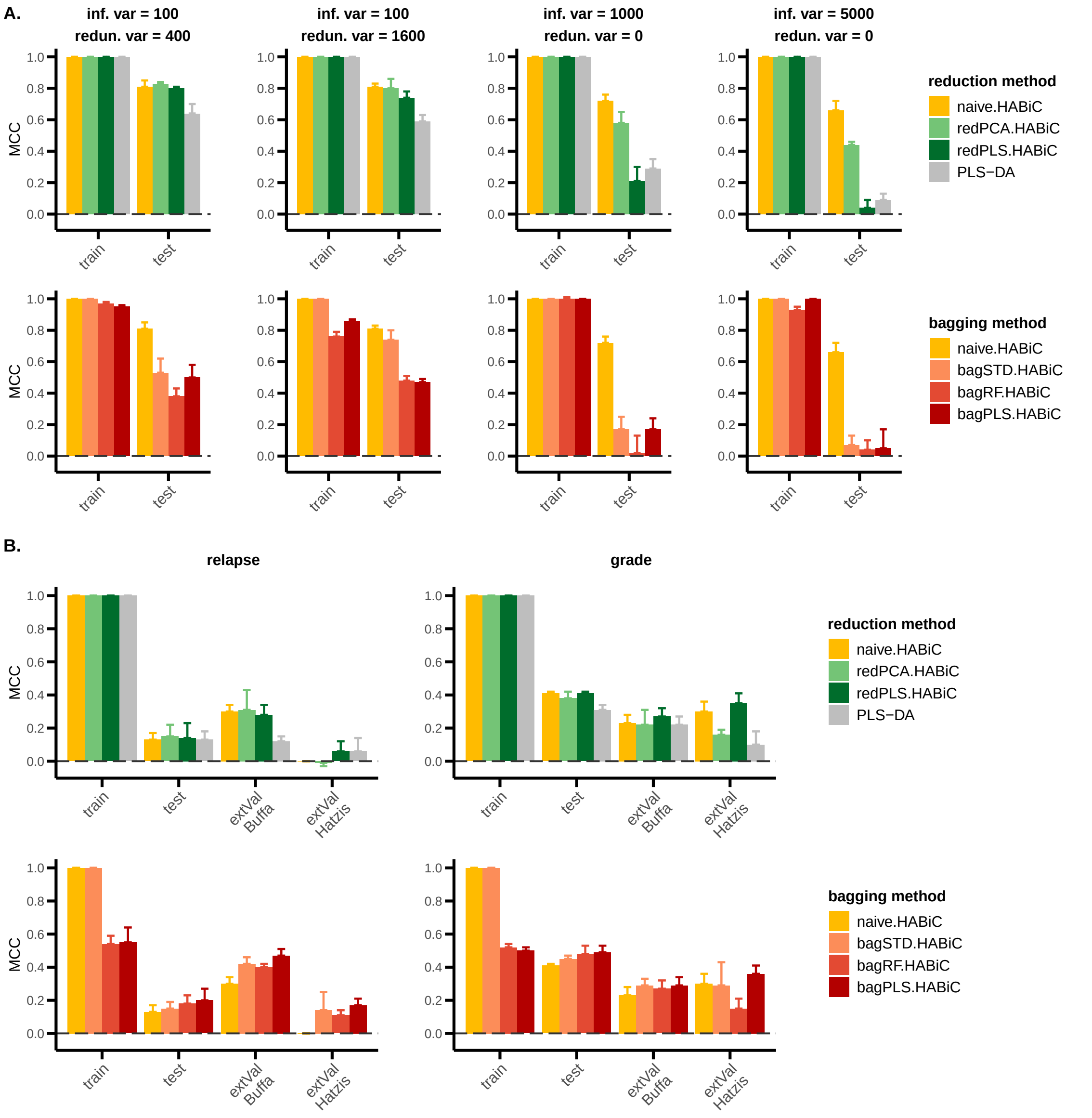

Supp. Figure 8: **MCC prediction performance of HABiC after regularization with dimension reduction or bagging approaches** in (A.) synthetic datasets with various combinations of informative and/or redundant variables and (B.) breast cancer transcriptomic datasets with relapse status or tumor histological grade to be predicted. Dimension reduction regularization was performed with either PCA (redPCA.HAbiC) or PLS-DA (redPLS.HAbiC), and PLS-DA prediction was used as control. Bagging regularization was performed either with the standard method (bagSTD) with $\sqrt{p}$ random variable selection, or with bagging with RF-based variable selection (bagRF), or with bagging with PLS-DA-based variable selection (bagPLS). Mean MCC values and standard deviations were calculated from 5-fold cross-validation. For real data, external validation (extVal) was also performed in two independent datasets (Buffa and Hatzis). Inf.var stands for informative variable, redun. var for redundant informative variable.

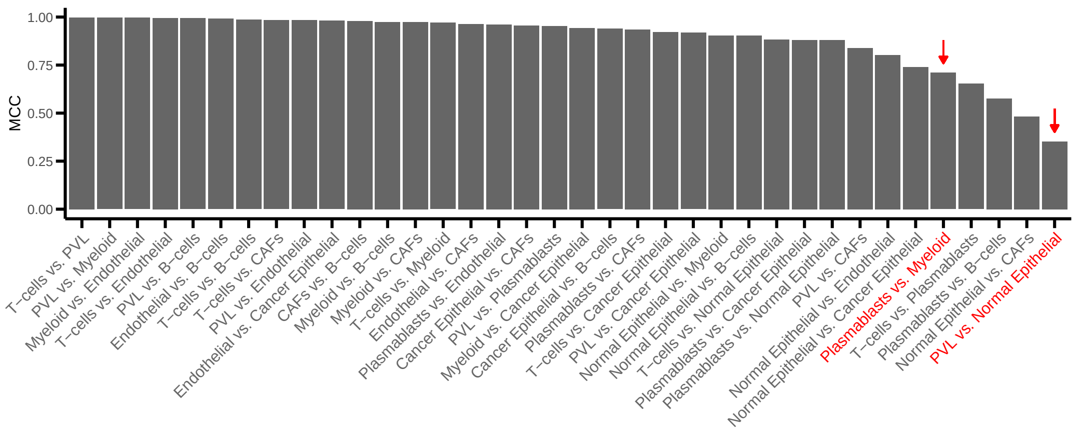

Supp. Figure 9: **RF prediction performance on an external validation cohort (Xu) in all possible cell type pairs based on annotated single-cell breast cancer transcriptomics.** Red arrows highlight the cell type pairs chosen for Figure 5 following this initial screening.
